# Dietary Antioxidants in the Mediterranean Diet

**DOI:** 10.3390/antiox10081213

**Published:** 2021-07-28

**Authors:** Alexandra Barbouti, Vlasios Goulas

**Affiliations:** 1 Department of Anatomy-Histology-Embryology, Faculty of Medicine, School of Health Sciences, University of Ioannina, 45110 Ioannina, Greece; 2 Department of Agricultural Sciences, Biotechnology and Food Science, Cyprus University of Technology, Lemesos 3036, Cyprus

Epidemiological studies performed during the second half of the previous century have correlated the diet that prevailed in the north shores of the Mediterranean basin with beneficial health effects, including reduced mortality risk and lower incidences of cardiovascular diseases [1]. This traditional dietary pattern was used as a basis to form the diet that is known worldwide as the “Mediterranean diet”, which is characterized by the high consumption of olive oil and plant foods such as fruits, vegetables, unprocessed cereals and legumes; a moderate consumption of dairy products, fish and wine; and a low consumption of red meat [2].

The beneficial health effects of the Mediterranean diet have been frequently attributed to antioxidants, compounds predominantly present in foods such as fruits, vegetables and olive oil [3]. It was assumed that dietary antioxidants protect cells and tissues from oxidations and prevent or delay the development of several diseases. It has to be emphasized, however, that the term “antioxidant”, although widely used in the literature, may cause misconceptions, most likely because antioxidants have a variety of applications (e.g., in industry, in food technology, in cosmetics, etc.). Moreover, a compound that can scavenge and neutralize free radicals in the test tube is not necessarily an effective antioxidant in living organisms [4,5]. According to the updated definition in the textbook of Halliwell and Gutteridge, an antioxidant is “any substance that delays, prevents or removes oxidative damage to a target molecule” [6] (p. 77).

The aim of this Special Issue of *Antioxidants* entitled “Dietary Antioxidants in the Mediterranean Diet” was to publish research papers and reviews addressing recent advances in the antioxidant composition of Mediterranean commodities and their potential biological activity. More specifically, it includes one review and seven original research papers.

Extra virgin olive oil (EVOO), the emblematic food of the Mediterranean diet, is widely recognized for its exceptional nutritional value. The healthy properties of EVOO have been attributed to its special fatty acid profile, and bioactive components [7] such as phenolics, which have been shown to have significant effect on human health [3,8]. In this Special Issue, the study of Lozano-Castellón et al. [9] presents the development and validation of a simple analytical method based on UHPLC coupled with electrospray ionization and MS/MS (UHPLC-ESI-MS/MS) for the analysis of the four major secoiridoids (which represent the major phenolic compounds in EVOO), able to coelute their isomers and achieve well-shaped peaks. It is noteworthy that the proposed method was validated following the Association of Official Agricultural Chemists (AOAC guidelines), and the matrix effect and recoveries were within satisfactory limits.

Native aromatic herbs also represent essential ingredients of the Mediterranean diet. They are used as culinary condiments to enhance the flavor, aroma and the color of many traditional Mediterranean dishes, or as herbal beverages. In has been reported that many herbs and spices possess a range of beneficial preventive health properties which have been attributed to their abundance of bioactive compounds [10]; therefore, the identification of phytochemical components is of great importance. Two studies in this Special Issue analyze the phenolic composition profile and the biological activities of *Thymus mastichina* extracts and rosemary infusions [11,12].

*Thymus mastichina* (also called mastic thyme, Spanish marjoram, or white thyme) is an endemic species from the Iberian Peninsula. It is widely used as a condiment because of its unique taste and aroma, and in folk medicine to treat several diseases [11]. The composition of its essential oil is well characterized; however, its phenolic profile is poorly investigated. The work by Taghouti et al. [11] describes the detailed phenolic composition of two *Thymus mastichina* extracts obtained by either exhaustive hydroethanolic extraction or aqueous decoction. Their results show that *Thymus mastichina* extracts contain high amounts of salvianolic acid derivatives, water-soluble compounds with a polyphenolic structure. The main salvianolic acid derivative was a salvianolic acid A isomer, which was identified for the first time in this plant. Additionally, a new salvianolic acid derivative was identified. Both hydroethanolic extract and aqueous decoction exhibited high free-radical scavenging capacity and the ability to inhibit the proliferation of two selected cancer cell lines (Caco-2, a human colon adenocarcinoma cell line and HepG2, a human hepatocellular carcinoma cell line) in a time- and dose-dependent manner.

Rosemary (*Rosmarinus officinalis* L., Lamiaceae family), another popular herb in the Mediterranean region, is also used as food seasoning and in folk medicine for the treatment of several disorders. The work of Peixoto et al. [12] examines the phenolic profile as well as the antioxidant (free-radical scavenging) potential of rosemary infusions. A total of forty-four phenolics (belonging to nine different groups: hydroxybenzoic acids, hydroxycinnamic acids, flavan-3-ols, flavanones, flavones, phenolic diterpenes, hydroxybenzaldehydes, coumarins, and pyranochromanones) were identified and, among them, seven were described for the first time in rosemary infusions. The *in vitro* antioxidant activity was assessed by DPPH^•^ scavenging ability, ferric reducing antioxidant power (FRAP) and oxygen radical absorbance capacity (ORAC) assays, and overall, rosemary infusions displayed strong antioxidant activity.

Cereals and cereal products are among the primary elements of the Mediterranean diet. Buckwheat (*Fagopyrum esculentum Moench*) is a cereal that has gained attention lately, as it is a rich source of protein, carbohydrates (starch), fiber, vitamins, and minerals. Buckwheat also possesses a high content of phenolic compounds, mainly rutin [13]. The fractionation of buckwheat seeds has been shown to concentrate certain components based on the varying proportion in their different parts. The objective of the work of Martín-García et al. [14] was to evaluate the suitability of sieving of buckwheat to produce flour fractions enriched with nutrients and bioactive substances such as phenolics. For that purpose, dehulled whole buckwheat grain was sieved, obtaining flour fractions with different particle sizes ranging from 215 µm to 45 µm. According to the results of this study, the buckwheat fraction with a 215 μm size was of most value in terms of nutritional components, as it was highly concentrated in free and bound phenolic compounds, protein, and ashes. The authors propose the use of sieving as an alternative green technology to obtain buckwheat flours naturally enriched with phenolic compounds and protein.

Except phenolic compounds, carotenoids are also a group of phytochemicals abundantly present in key components of the traditional Mediterranean diet. They give fruits and vegetables yellow to reddish shades and are recognized as playing important roles in decreasing the risk of several diseases [15]. Some carotenoids contribute to dietary vitamin A, but they are also potent antioxidants. Due to their lipophilic nature, co-ingestion with fat appears to increase their intestinal absorption, and hence their plasma concentrations. Although it has been proposed that fat intake increases their bioavailability, further research is needed in order to investigate whether this association is linear or if it plateaus at some point. A cohort study by Marhuenda-Muñoz et al. [16] examined the association of fruit and vegetable consumption and fat intake, with plasma concentrations of carotenoids in an older Mediterranean population with metabolic syndrome. The study population was categorized into four groups according to their self-reported consumption of fruits/vegetables and fat. As expected, carotenoids systemic concentrations were greater in high consumers of fruits and vegetables, than in low consumers of these foods. However, when the groups were separated according to dietary fat intake, circulating concentrations of carotenoids seemed to decrease when total fat intake was very high. These results suggest that the high consumption of fruits and vegetables is associated with higher systemic levels of total carotenoids, particularly when fat intake is low-to-moderate rather than very high.

A review paper by Barbouti et al. [17], propose that dietary bioactive compounds present in the Mediterranean-type diet protect against oxidative stress-induced cellular ageing by acting as iron chelating compounds, rather than free-radical scavenges. Among several theories that have been proposed to explain the molecular basis of ageing, the so-called “free-radical theory of ageing” [18], has gained widespread acceptance. It proposes that organismal ageing is predominantly the consequence of accumulated oxidative damage to cells and tissues, which comes from the deleterious side attacks of free radicals and other reactive oxygen species produced even during normal aerobic metabolism. In conditions of ongoing oxidative stress, the ability of cells to repair their damaged constituents reach saturation, and over-oxidized non-repairable “waste material” accumulates inside the cells. This chemically undefined material conventionally called “lipofuscin” is a common hallmark of ageing (senescent) cells [17]. A necessary requirement for the intracellular generation of highly reactive free radicals is the presence of “labile iron” [19]. The former represents a small and finely adjusted portion of unshielded iron that is able to participate in damaging free-radical generating reactions known as Fenton reactions [19]. It has been shown that the chelation or re-distribution of intracellular labile iron by pharmacological agents may diminish oxidative stress-induced cell damage and cell death [20,21]. Interestingly, typical plant-based foods of the Mediterranean diet contain a plethora of iron-chelating phytonutrients. For instance, it has been reported that phenolics with an ortho-dihydroxyl group protect human cell lines against oxidative stress conditions by chelating intracellular iron [3,22,23,24,25]. Based on these considerations, the review article by Barbouti et al. [17] proposes that iron-chelating bioactive compounds contained in the Mediterranean-type diet represent crucial factors that may prevent lipofuscin formation and, consequently, cellular ageing. In order to exhibit cytoprotective effects, these diet-derived phytochemicals must be able to penetrate cellular membranes and chelate cellular labile iron, so as to diminish undesirable oxidations in critical cellular constituents.

Undoubtedly, the plethora of bioactive constituents of Mediterranean diet foods are, at least in part, responsible for the observed health benefits, and further research is needed to elucidate their mechanism(s) of action [5]. Moreover, agricultural wastes and by-products contain similar functional ingredients with the potential for producing value-added products suitable for food industrial, pharmaceutical or cosmetic applications [26]. Additionally, the effective valorization of these residual materials can efficiently help in decreasing the unwanted environmental pollution.

A work by Goulas et al. [27] examines the potential of sun-dried grape pomace to be used as a multi-functional ingredient for herbal infusion. Wine and by-products are essential elements of the Mediterranean diet and are considered as a rich source of bioactive compounds with various health effects. Therefore, the utilization of sun-dried grape pomace to produce functional infusions is an alternative strategy to promote the sustainability of winemaking and to create a novel product which shares the antioxidant components of grapes. Goulas et al. [27] describe the optimization of brewing parameters such as the ratio of water to grape pomace powder (40–200 mL g^−1^), infusion time (3–15 min) and temperature (55–95 °C) to produce a multi-functional infusion using multiple response optimization. Overall, the present work describes a promising strategy for the re-use of sun-dried grape pomace as a functional ingredient of infusion.

Olive tree-related extracts (such as olive tree leaves or olive mill waste extracts) are also a valuable source of bioactive compounds. For instance, only 2% of olive fruit phenolics are transferred to olive oil during the production process, whereas 98% of them are retained in the cake [28]. In the work by Hernáez et al. [29] several phenolic extracts from olive-tree related products (olive leaves, olive oil solid waste and olive oil) were examined *ex vivo* for their antioxidant, vasoactive, anti-inflammatory and antithrombotic capacities. Subsequently, extracts with higher scores were mixed and re-examined in order to assess which of the selected combinations exhibited the most favorable profile in terms of the aforementioned bioactivities. Their results showed that a combination of phenolic compounds that was rich in 3,4-dihydroxyphenylglycol, hydroxytyrosol, and oleuropein showed the best dose-adjusted in vitro properties, as it scored the highest antioxidant, vasoactive and antithrombotic properties.

In conclusion, the key ingredients of the Mediterranean diet, such as extra virgin olive oil, fruits and vegetables, grains, wine and herbs, contain numerous compounds that act in a beneficial way regarding the development of various chronic diseases. Predominantly present in the aforementioned foods are strong antioxidants of the free-radical scavenging type, which have been assumed to protect against oxidative stress and related diseases. Notably, accumulating evidence indicates that several diet-derived compounds can exert also specific biological functions which are unrelated with their intrinsic reducing capacity. Therefore, further research is needed to unveil the molecular mechanisms by which dietary compounds present in Mediterranean diet exert their beneficial health effects.
